# Sequential modulation of distractor-interference produced by semantic generalization of stimulus features

**DOI:** 10.3389/fpsyg.2014.01271

**Published:** 2014-11-14

**Authors:** Mike Wendt, Aquiles Luna-Rodriguez, Thomas Jacobsen

**Affiliations:** Experimental Psychology Unit, Helmut Schmidt University/University of the Federal Armed Forces HamburgHamburg, Germany

**Keywords:** conflict adaptation, Gratton effect, feature integration, semantic generalization, episodic memory

## Abstract

Sequential modulations of distractor-related interference (i.e., reduced congruency effect after incongruent as compared to congruent predecessor trials, a.k.a. Gratton effect) have been taken to reflect conflict-induced attentional focusing. To dismiss an alternative interpretation based on integration and retrieval of low-level features, it is important to exert experimental control of stimulus and response feature sequences. This has been achieved by considering only trials associated with complete feature changes. Furthermore, distractors from two different perceptual dimensions, such as stimulus location and shape, have been combined in the same experiment to investigate the question of specificity vs. generality of conflict adaptation. With this method feature sequence control can be exerted, in principle, without disregarding data from feature repetition trials. However, such control may be insufficient when the distractor dimensions overlap semantically. In two experiments we found evidence consistent with the assumption that semantic generalization of stimulus features, such as between a stimulus presented at a left-sided location and a stimulus shape pointing to the left, may yield a between-dimension Gratton effect. These findings raise doubts about inferring generalized attentional conflict adaptation when semantically related distractor dimensions are used.

## Introduction

When people have to base the selection of a response on a specific stimulus object or feature, processing selectivity is often incomplete in the sense that other stimulus features, which are formally not needed for successful task performance, receive cognitive processing up to a level of behavioral relevance. This can be seen in slower and/or less accurate performance on trials in which an irrelevant stimulus feature (henceforth *distractor*) is associated with an incorrect response (henceforth *incongruent* condition) compared to with the same response as the target feature (henceforth *congruent* condition). This congruency effect has been found to be reduced after incongruent as compared to after congruent predecessor trials, a sequential modulation often referred to as the *Gratton effect*. The Gratton effect has been observed in a variety of different tasks, such as the Stroop task (e.g., Kerns et al., 2004), the Eriksen flanker task (e.g., Gratton et al., 1992), the Simon task (e.g., Notebaert et al., 2001; Wühr and Ansorge, 2005), and different versions of priming tasks (e.g., Kunde and Wühr, 2006; Hazeltine et al., 2011). The dominant interpretation of the Gratton effect implies the assumption of increased focusing of attention on the target stimulus dimension after processing a high-interference stimulus event (Botvinick et al., 2001; Blais et al., 2007; Verguts and Notebaert, 2008, 2009; see Gratton et al., 1992, for a related account).

An alternative view to this attentional adaptation account was put forward by Hommel et al. (2004), see Mayr et al. (2003), for a related idea. Based on Hommel's (Hommel, 1998; Hommel and Colzato, 2004) feature integration theory, the Gratton effect is assumed to result from the retrieval of stimulus and response features previously bound together in episodic memory. According to feature integration theory, stimulus and response features that co-occur close in time are integrated in transient memory episodes, referred to as *event files*. Activation of an item of an event file due to a match with current perceptual input or response demands is assumed to co-activate the other feature(s) of the event file, and mismatches between co-activated representations and current perceptual input or response demands are assumed to interfere with response selection. Therefore, feature integration theory predicts performance impairments on *partial repetition trials* (i.e., on trials associated with repetition of one stimulus or response feature and alternation of another one from the preceding trial) compared to complete feature repetition or alternation trials.

The possible role of stimulus and response feature sequences for the Gratton effect becomes apparent if one looks at the sequences of congruency levels in standard interference tasks. Consider a typical Simon task, in which participants perform a binary classification of a non-spatial target feature, such as judging whether a given stimulus is black or white, by pressing one of two response keys. On each trial, the stimulus is presented in one of two possible locations, each of which spatially corresponds to the location of one of the responses, a left one and a right one, say. A given trial is congruent if stimulus and response locations fall on the same side and incongruent if stimulus and response locations fall on opposite sides. As can be seen in Table 1, with such an arrangement there is a confound between the sequence of congruency levels on the one hand and the sequence of stimulus and response locations on the other. Specifically, the congruency level repeats if and only if either both the target/response and the stimulus location repeat or if all these features alternate. Conversely, alternations of the congruency level are bound to trial transitions with repetition of either the stimulus location or the target/response and alternation of the other feature(s). Given this confound, feature integration theory predicts facilitated performance on congruency level repetition trials (i.e., congruent → congruent or incongruent → incongruent) compared to congruency level alternation trials (i.e., congruent → incongruent or incongruent → congruent), and thus a Gratton effect.

**Table 1:**
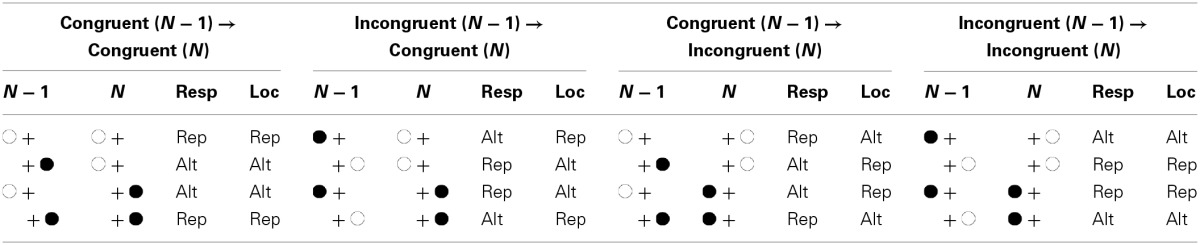
**Stimulus displays in trials *N* − 1 and *N*, sequence of the response, and sequence of the stimulus location as a function of congruency on trial *N* − 1 and trial *N***.

*White circles indicate left-sided responses, black circles indicate right-sided responses. + indicates the center of the display. Resp, Response; Loc, (Stimulus) Location, Rep, Repetition, Alt, Alternation*.

Previous research has tried to deconfound the sequence of congruency levels and the sequences of stimulus and response features. One approach is characterized by using a larger number of stimuli and responses and confining the sequential congruency analysis to trials in which all discriminative stimulus and response features differ from the preceding trial. Applying this approach bears the risk of associating congruent and incongruent stimuli with different degrees of distractor-target or distractor-response contingencies, thereby making it possible to account for a Gratton effect in terms of contingency level switch costs rather than conflict adaptation (Schmidt and De Houwer, 2011; Mordkoff, 2012; Schmidt, 2013). Indeed, previous studies that controlled contingencies by using a four-choice task and choosing both the target and the distractor completely randomly on each trial have failed to replicate the Gratton effect (Schmidt and De Houwer, 2011; Mordkoff, 2012). Some recent studies, however, successfully observed Gratton effects under conditions of controlled feature sequences and contingencies. This was achieved by dividing a four-choice task into a pair of two-choice tasks involving distinct sets of targets and distractors. With this arrangement, contingencies are unbiased when congruent and incongruent trials are administered with a probability of 50% each. Trial-to-trial feature repetitions were prevented by alternating between the two tasks on every trial (Kim and Cho, 2014, Experiment 1; Schmidt and Weissman, 2014; Weissman et al., 2014) or by eliminating all data from trials associated with a feature repetition from the analysis (Freitas et al., 2007, Experiment 1; Freitas and Clark, 2014, Experiment 1). Although Gratton effects have thus been obtained in the absence of feature repetitions and biased contingencies, the evidence so far is confined to a particular procedure of grouping targets and distractors into distinct two-choice tasks, devoid of any featural overlap. Future research is needed to clarify the factors underlying the superiority of this “task-splitting procedure.”

A different methodological approach that has been applied in research on sequential conflict adaptation includes the presentation of distractors that belong to two different perceptual dimensions. Examining the congruency effect regarding one dimension as a function of the preceding congruency level regarding the other dimension (henceforth, if a sequential modulation is obtained, *between-dimension Gratton effect*) speaks to the question of specificity vs. generality of conflict adaptation mechanisms (for an overview, see Egner, 2008). As will be shown in detail below, the standard experimental set-ups used for this purpose nicely control for the feature sequence confound laid out above. Moreover, with a standard experimental design involving binary target and distractor sets and random choice of both the target and the distractor(s) on each trial, distractor-target/response contingencies are constantly unbiased, irrespective of the congruency level sequence.

Empirically, most studies which combined distractors from different perceptual dimensions failed to yield between-dimension Gratton effects (e.g., Egner et al., 2007; Fernandez-Duque and Knight, 2008; Notebaert and Verguts, 2008, condition 2; Funes et al., 2010a,b; Akçay and Hazeltine, 2011; Lee and Cho, 2013; Stürmer et al., 2005; Verbruggen et al., 2005; Wendt et al., 2006; Schlaghecken et al., 2011; Torres-Quesada et al., 2013, 2014)^1^.

In some studies, however, between-dimension Gratton effects were successfully obtained (e.g., Kunde and Wühr, 2006; Freitas et al., 2007; Notebaert and Verguts, 2008, condition 1; Freitas and Clark, 2014). For illustration, consider Experiment 2 of Kunde and Wühr (2006). Participants responded to the left or right direction of a stimulus arrow with spatially corresponding key presses. The arrow occurred randomly on the left or on the right side of the screen and was preceded by a prime stimulus (a smaller arrow) in the same location, which could also point to the left or right. We shall refer to (mis)match between arrow direction and response location as *direction-(in)congruency* and to (mis)match between stimulus and response location as *location-(in)congruency*. In addition to within-dimension Gratton effects (i.e., a reduced direction-based congruency effect after direction-incongruent trials and a reduced location-based congruency effect after location-incongruent trials), Kunde and Wühr observed—albeit smaller—reductions of location- and direction-based congruency effects after incongruent trials regarding the other distractor dimension.

Table 2 shows the congruency level sequences and the sequences of distractor and response features under such circumstances^2^. As can be seen in Table 2, unlike the sequence of congruency levels regarding the same distractor dimension, the sequence of congruency levels regarding different distractor dimensions is not confounded with the sequences of the distractor stimulus features (i.e., arrow direction and stimulus location), the response, or the combination of these features. That is, unlike within-dimension congruency level repetitions and alternations, between-dimension congruency level repetitions and alternations are associated with the same amount of conjoined and partial stimulus and response feature repetitions and alternations.

**Table 2:**
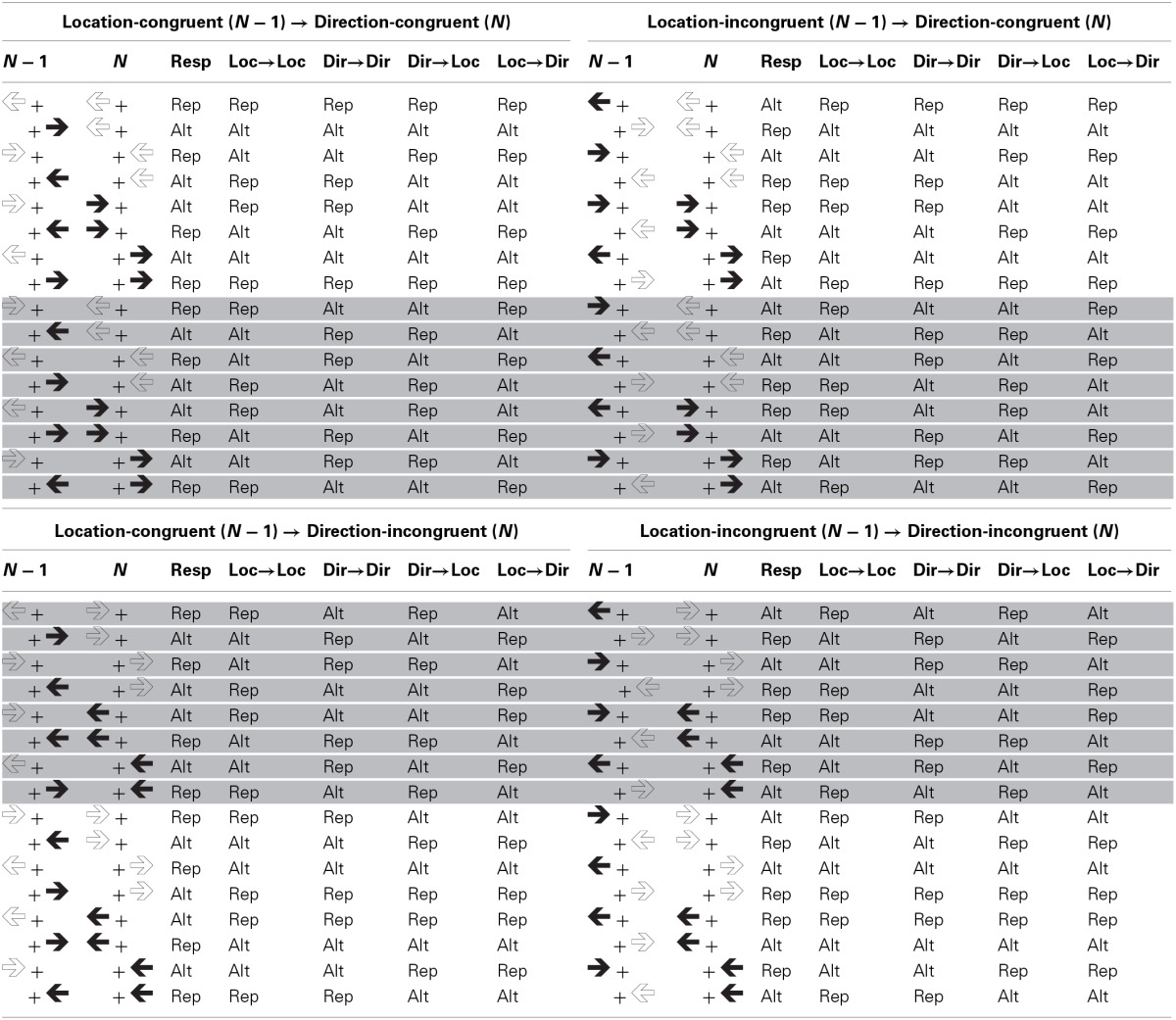
**Stimulus displays in trials *N* − 1 and *N*, sequences of the response, and sequences of concrete (location-to-location and direction-to-direction) and abstract (direction-to-location and location-to-direction) distractor features as a function of direction-congruency on trial *N* and location-congruency on trial *N* − 1**.

*Arrows represent directional information in the distractor stimulus feature. The target stimulus feature is indicated by color: White indicates a left-sided response, black indicates a right-sided response. + indicates the center of the display. Unshaded and shaded areas indicate abstract feature sequence match and mismatch trials, respectively (see text for explanation). Resp, Response; Dir, (Arrow) Direction; Loc, Stimulus Location; Rep, Repetition; Alt, Alternation*.

Notwithstanding this independence, the sequence of congruency levels may be confounded with more abstract stimulus features at least for certain combinations of distractor dimensions. In the current study, we focus on one particular kind of abstract features inherent in manipulations involving two distractor dimensions that are *semantically related*. Consider again the example depicted in Table 2. In this example both stimulus location and arrow direction overlap semantically in that each can take one of two values, that is, left or right. Such semantic overlap offers the possibility to account for a between-dimension Gratton effect in terms of feature integration theory, if one assumes semantic generalization of stimulus features in the sense that a stimulus presented on one side, the left say, tends to activate a left-pointing arrow integrated in a previously formed event file and vice versa. This interpretation becomes apparent if one looks at the sequences of left/right features occurring in different formats (i.e., stimulus location and arrow direction) on consecutive trials. As can be seen in the “Location → Direction” and “Direction → Location” columns of Table 2, between-dimension congruency level alternations are associated with more partial repetitions regarding such abstract left/right feature-response sequences than between-dimension congruency level repetitions. Consider the case that a direction-congruent trial follows a location-incongruent trial (i.e., between-dimension congruency level alternation). If the response repeats a left-sided stimulus location is followed by a prime arrow pointing to the right or a right-sided stimulus location is followed by a prime arrow pointing to the left (i.e., abstract feature alternation, see column “Location → Direction”). If the response alternates a left-sided stimulus location is followed by a prime arrow pointing to the left or a right-sided stimulus location is followed by a prime arrow pointing to the right (i.e., abstract feature repetition). Assuming semantic generalization, this contingency could impair performance, possibly leading to a reduced direction-based congruency effect after a location-incongruent trial, hence a between-dimension Gratton effect.

This *semantic generalization hypothesis* could also explain why in Kunde and Wühr's (2006) experiment within-dimension Gratton effects were more pronounced than between-dimension Gratton effects. Such a difference in effect strength can be expected for two reasons. First, activation of a feature in an event file should be triggered more reliably or more strongly by perception of an identical rather than a semantically related feature. Second, the confound of between-dimension congruency level sequences and abstract feature sequences is less complete than the confound of within-dimension congruency level sequences and concrete feature sequences, as will be elaborated in the following paragraph.

To gain evidence about semantic generalization of stimulus features that belong to different distractor dimensions it is instructive to look at the relationship of congruency level sequences and abstract feature sequences in more detail. Inspection of Table 2 shows that, for half of the trials, the location-direction sequence matches the direction-location sequence in the sense that when the abstract left/right feature repeats regarding the location-direction transition, it also repeats regarding the direction-location transition. Also, when the abstract left/right feature alternates regarding the location-direction transition, it also alternates regarding the direction-location transition (see top and bottom quarters of Table 2). For example, in line 3 of Table 2 a left-sided stimulus location in trial *N* − 1 is followed by an arrow prime pointing to the left in trial *N* while at the same time an arrow prime pointing to the right in trial *N* − 1 is followed by a right-sided stimulus location in trial *N*. These sequences mismatch on the other half of trials (see (shaded) middle quarters of Table 2). We denote the former trials as *abstract (feature sequence) match* trials and the latter trials as *abstract (feature sequence) mismatch* trials.

It is important to note that whereas on abstract match trials the sequence of the response either matches or mismatches both abstract feature sequences (i.e., location-to-direction and direction-to-location), on abstract mismatch trials the sequence of the response matches one of the abstract feature sequences and mismatches the other one. Thus, on abstract match trials, a conjoined repetition or alternation of the response and the abstract left/right feature regarding one between-dimension transition (e.g., location-direction) is always associated with a conjoined repetition or alternation of the response and the abstract left/right feature regarding the reversed transition (i.e., direction-location). And a partial repetition regarding the response and the abstract left/right feature regarding one between-dimension transition is always associated with a partial repetition regarding the response and the abstract left/right feature regarding the reversed between-dimension transition. In contrast, on abstract mismatch trials, whenever there is a conjoined repetition or alternation of the response and the abstract left/right feature regarding one between-dimension transition, there is a partial repetition regarding the response and the abstract left/right feature regarding the reversed between-dimension transition.

As a consequence of this contingency, on abstract match trials both semantic sequence effects should work in the same direction, whereas on abstract mismatch trials they should work in opposite directions. Assuming that semantic location-direction transitions and direction-location transitions yield effects of comparable strength—that is, a stimulus location integrated in an event file is activated by perceiving a corresponding arrow direction to roughly the same amount as an arrow direction integrated in an event file is activated by perceiving a corresponding stimulus location—semantic sequence effects should add up to zero on abstract feature sequence mismatch trials. On the corollary assumption of comparable effect strength, semantic generalization should therefore yield a between-dimension Gratton effect selectively on abstract match trials and an absence thereof on abstract mismatch trials. The semantic generalization hypothesis thus predicts between-dimension Gratton effects on abstract match trials and, assuming comparable efficacy of semantic generalization for both dimension transition directions, the absence of a between-dimension Gratton effect on abstract mismatch trials.

## Experiment 1

In the first experiment of the current study, we explored the role of semantic generalization for between-dimension Gratton effects by combining stimulus location and pointing direction of the stimulus shape, each of which could vary between the values left and right, as distractor dimensions, thereby producing the contingencies displayed in Table 2. Participants classified the color of stimulus arrows, which pointed either to the left or to the right and occurred either to the left or to the right of the screen center. A pilot experiment yielded location-based interference (i.e., a Simon effect) but no main effect of direction-based congruency. A possible explanation for this result is that participants did not sufficiently code the stimulus arrows as pointing to the left or to the right because arrow direction was never relevant throughout the experiment. To increase the likelihood of left/right coding of the arrow direction, we inserted blocks of trials in which participants responded to the direction of the arrows rather than to their colors in Experiment 1.

### Method

#### Participants

Two female and 13 male students of the Helmut-Schmidt-University/University of the Federal Armed Forces Hamburg participated in exchange for partial course requirements. They ranged in age from 19 to 28 years. The experiments of the current study were conducted in accordance with the ethical guidelines of the German Psychological Society (Deutsche Gesellschaft für Psychologie) and the Declaration of Helsinki of the World Medical Association. Formal ethics approvals for the described kind of research are not required by the guidelines of the German Psychological Association or the World Medical Association.

#### Apparatus and stimuli

**Figure 1:**
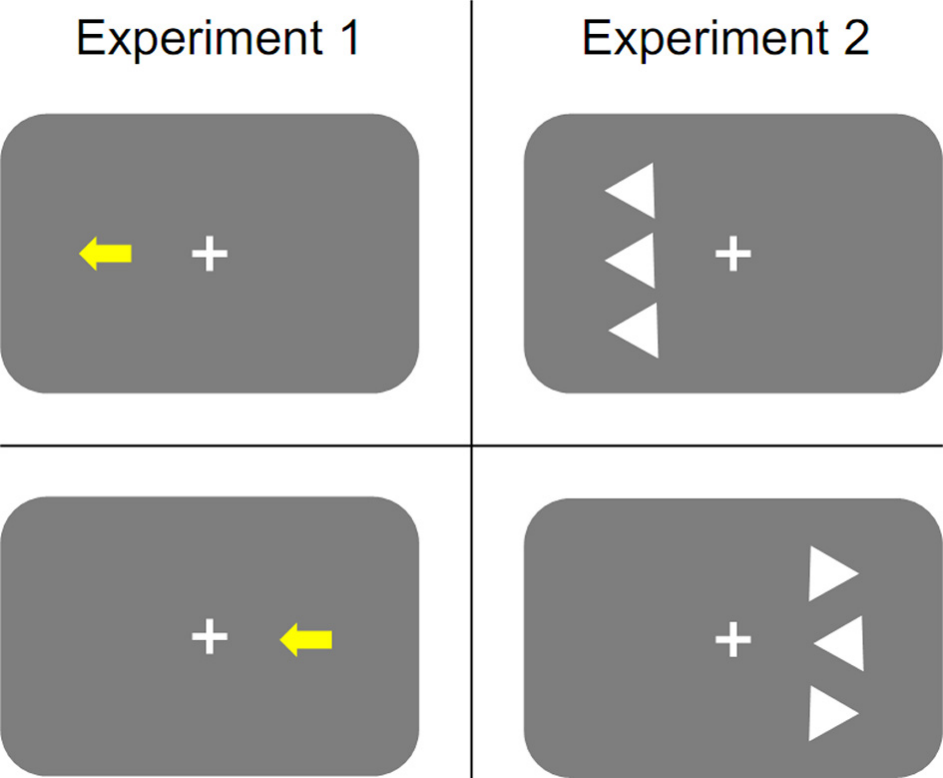
**Schematic examples of stimulus displays used in Experiment 1 (left panel) and Experiment 2 (right panel)**. Regarding Experiment 1, for participants that responded to yellow arrows with the left-sided response and to blue arrows with the right-sided response the top row depicts a stimulus of a location-congruent/direction-congruent trial and the bottom row depicts a stimulus of a location-incongruent/direction-congruent trial. For participants that responded to yellow arrows with the right-sided response and to blue arrows with the left-sided response the top row depicts a stimulus of a location-incongruent/direction-incongruent trial and the bottom row depicts a stimulus of a location-congruent/direction-incongruent trial. Regarding Experiment 2, the top row depicts a stimulus of a location-congruent/direction-congruent trial, and the bottom row depicts a stimulus of a location-incongruent/direction-congruent trial.

The stimuli were presented on a 17-in. monitor with a refresh rate of 60 Hz. A dark gray background was used. Stimuli were arrows pointing either to the left or to the right, which extended 3.0 cm horizontally and 2.5 cm vertically. As mentioned above, we inserted blocks of trials, in which participants responded to arrow direction, amongst the experimental blocks, in which stimulus color had to be judged. We refer to the former blocks as intermediate blocks, and to the latter blocks as critical blocks. In the critical blocks, arrows were presented in either blue or yellow color and occurred either 2.4 cm to the left or to the right of the screen center (nearest edge) on the horizontal midline. In the intermediate blocks the arrows were white stimuli and occurred in the screen center. Two response keys were used. They were located on an external keyboard and extended 1.0 × 1.0 cm. One key was located 4.0 cm to the left and the other 4.0 cm to the right of the keyboard's saggital midline, which was placed perpendicular to the screen and aligned with the screen center. The left response key was pressed with the index or middle finger of the left hand; the right response key was pressed with the index or middle finger of the right hand. During an experimental block the fingers remained on the keys. Regarding the critical blocks, odd-numbered participants were instructed to press the left key for yellow and the right key for blue. This assignment was reversed for even-numbered participants. In the intermediate blocks, participants were to respond to the direction of the arrow with the spatially corresponding key press. Figure 1, left panel, depicts schematic examples of stimulus displays used in different conditions of the critical blocks.

#### Procedure

Participants sat approximately 50 cm from the computer screen. Each trial began with the presentation of a fixation cross in the center of the screen (0.3 × 0.3 cm). After a period of 500 ms, an arrow stimulus was presented. In the critical blocks, the color (blue vs. yellow), the location (left vs. right), and the direction (left vs. right) of the arrow were chosen randomly on each trial. In the intermediate blocks, arrow direction was again chosen randomly on each trial. Participants were instructed to classify the stimulus by pressing the assigned response key as quickly as possible while avoiding errors. Immediately after a response key was pressed, the stimulus and the fixation cross disappeared from the screen. In case of a correct response the next trial started 500 ms after the response with the presentation of the fixation cross. In case of an incorrect response, the German word “falsch” (“incorrect”) occurred for 800 ms slightly below the screen center. Then the trial was repeated with an identical stimulus. Repetitions of incorrect trials were not counted as trials (and not subjected to the statistical analyses).

At the beginning of the experiment participants received written instructions. After a practice block of 30 trials, which was structurally identical to the critical blocks, participants were presented with 24 critical blocks of 35 trials each. In advance of each critical block, an intermediate block of 16 trials was administered. Only critical blocks were subjected to the statistical analysis. Between blocks, participants were allowed to rest for some time. A complete session took between 40 and 45 min.

### Results

The first three trials of each critical block were considered “warm-up” trials and not analyzed. Furthermore, we excluded data from the first two trials following an error as well as RTs associated with an incorrect response or smaller than 200 ms or larger than 1200 ms. 0.2% of the data were eliminated by excluding RT outliers.

Two sets of analysis, an overall analysis of within-dimension and between-dimension Gratton effects, and a semantic feature sequence analysis, were conducted. In the overall analysis, trials were classified depending on location-congruency and direction-congruency on the current (*N*) and the preceding (*N* − 1) trial. To investigate the semantic generalization hypothesis, we analyzed between-dimension sequential congruency effects depending on match vs. mismatch of the abstract feature sequences.

#### Overall analysis

Analyses of Variance (ANOVAs) with repeated measures on the factors direction-congruency on the current trial (congruent, incongruent), location-congruency on the current trial (congruent, incongruent), direction-congruency on the preceding trial (congruent, incongruent), and location-congruency on the preceding trial (congruent, incongruent) were conducted on the mean RTs and error proportions.

Responding took longer when the current trial was associated with an incongruent as compared to a congruent arrow direction (378 vs. 372 ms), *F*_(1, 14)_ = 5.8; *p* < 0.04; *MSE* = 310.1, and when the current trial was associated with an incongruent as compared to a congruent stimulus location (388 vs. 362 ms), *F*_(1, 14)_ = 39.1; *p* < 0.01; *MSE* = 1034.2. Within-dimension Gratton effects reliably occurred for both distractor dimensions. The direction-based congruency effect was reduced from 17 to -6 ms after a direction-incongruent trial as compared to a direction-congruent trial, *F*_(1, 14)_ = 7.9; *p* < 0.02; *MSE* = 858.7, and the location-based congruency effect was reduced from 57 to −4 ms after a location-incongruent trial as compared to a location-congruent trial, *F*_(1, 14)_ = 125.6; *p* < 0.01; *MSE* = 443.5. Regarding between-dimension sequential modulations, the direction-based congruency effect was reduced after a location-incongruent trials as compared to a location-congruent trials (0 vs. 11 ms), *F*_(1, 14)_ = 6.9; *p* < 0.02; *MSE* = 257.9. Likewise, the location-based congruency effect was reduced after direction-incongruent as compared to after direction-congruent trials (24 vs. 29 ms), *F*_(1, 14)_ = 4.7; *p* < 0.05; *MSE* = 87.2.

In the error analysis, the main effect of direction-congruency failed to reach significance (4.1 vs. 4.9%, for congruent and incongruent trials, respectively), *F*_(1, 14)_ = 3.2; *p* = 0.10; *MSE* = 0.00116. Errors were more frequent, however, when the current trial was associated with an incongruent as compared to a congruent stimulus location (5.6 vs. 3.4%), *F*_(1, 14)_ = 6.9; *p* < 0.03; *MSE* = 0.00434. In addition, an incongruent arrow direction on the preceding trial reduced errors from 4.9 to 4.1%, *F*_(1, 14)_ = 5.2; *p* < 0.04; *MSE* = 0.00061. Again, within-dimension Gratton effects occurred for both dimensions: The direction-based congruency effect was reduced from 1.9 to −0.3% after a direction-incongruent trial compared to a direction-congruent trial, *F*_(1, 14)_ = 8.4; *p* < 0.02; *MSE* = 0.00098, and the location-based congruency effect was reduced from 7.4 to -3.0% after a location-incongruent trial compared to a location-congruent trial, *F*_(1, 14)_ = 25.4; *p* < 0.01; *MSE* = 0.00630. By contrast, no between-dimension sequential modulation occurred, both *F*s_(1, 14)_ < 1.

#### Semantic generalization effects

**Figure 2:**
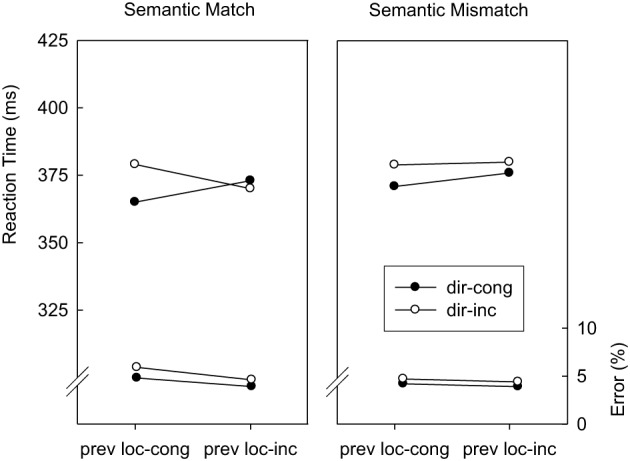
**Mean reaction times and error percentages in Experiment 1 as a function of direction-congruency on the current trial (dir-cong, dir-inc), location-congruency on the preceding trial (prev loc-cong, prev loc-inc), and correspondence of the abstract feature sequences (match, mismatch)**.

***Location-congruency (*N* − 1) → direction-congruency (N)***. Trials were classified as a function of direction-congruency in trial *N* (congruent, incongruent), location-congruency in trial *N* − 1 (congruent, incongruent), and abstract correspondence (match, mismatch). Figure 2 displays mean RTs and error percentages for these data. Regarding RTs, the main effect of direction-congruency as well as the interaction with the location-congruency level of the preceding trial were replicated from the overall analysis, *F*_(1, 14)_ = 4.9; *p* < 0.05; *MSE* = 207.4, and *F*_(1, 14)_ = 5.7; *p* < 0.04; *MSE* = 139.6, respectively. As predicted by the semantic generalization hypothesis, this was modulated by the abstract correspondence, *F*_(1, 14)_ = 10.3; *p* < 0.01; *MSE* = 31.1. Whereas on abstract match trials, the direction-based congruency effect amounted to 14 ms after location-congruent trials and −3 ms after location-incongruent trials, abstract mismatch trials were associated with 8 and 4 ms of direction-based interference after location-congruent and location-incongruent trials, respectively. The error data yielded no significant effects.

**Figure 3:**
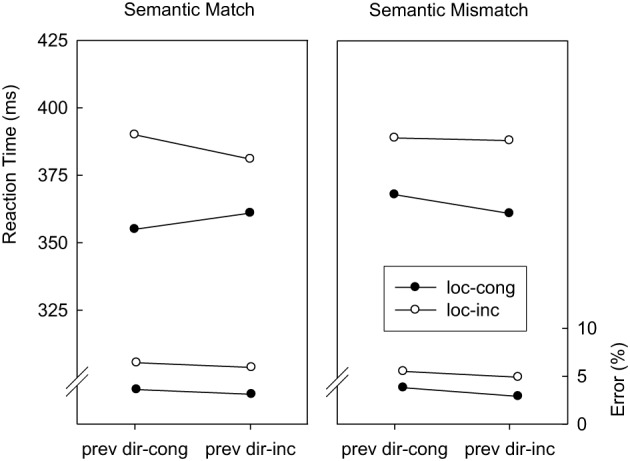
**Mean reaction times and error percentages in Experiment 1 as a function of location-congruency on the current trial (loc-cong, loc-inc), direction-congruency on the preceding trial (prev dir-cong, prev dir-inc), and correspondence of the abstract feature sequences (match, mismatch)**.

***Direction-congruency (*N* − 1) → Location-congruency (N)***. Trials were classified as a function of location-congruency in trial *N* (congruent, incongruent), direction-congruency in trial *N* − 1 (congruent, incongruent), and abstract correspondence (match, mismatch). Figure 3 displays mean RTs and error percentages for these data. Regarding RTs, the main effect of location-congruency as well as the interaction with direction-congruency of the preceding trial were replicated from the overall analysis, *F*_(1, 14)_ = 35.8; *p* < 0.01; *MSE* = 564.0, and, *F*_(1, 14)_ = 4.9; *p* < 0.05; *MSE* = 29.6, respectively. In line with the semantic generalization hypothesis, reduction of the location-based congruency effect after direction-incongruent trials occurred selectively on abstract match trials, *F*_(1, 14)_ = 6.2; *p* < 0.03; *MSE* = 131.4. Specifically, on abstract match trials, the location-based congruency effect amounted to 35 ms after direction-congruent trials and 20 ms after direction-incongruent trials, whereas on abstract mismatch trials the location-based congruency effect was 21 ms after direction-congruent trials and 28 ms after direction-incongruent trials. The error data yielded only a significant main effect of location-congruency, *F*_(1, 14)_ = 7.4; *p* < 0.02; *MSE* = 0.00221.

### Discussion

Experiment 1 replicated previously found sequential modulations of location-based interference and of direction-based interference. Although these within-dimension Gratton effects are in line with the conflict adaptation hypothesis, they can also be accounted for in terms of distractor-target or distractor-response feature integration because of the complete confound of the sequences of congruency levels and the sequences of specific distractor and target/response conjunctions. In addition, replicating the finding of Kunde and Wühr (2006), both direction- and location-based interference were reduced after an incongruent compared to after a congruent distractor of the other stimulus dimension. In light of evidence suggesting different time courses of interference effects elicited by location-based and symbolic spatial distractors (Pellicano et al., 2009), these findings are remarkable from a conflict adaptation perspective that assumes that conflict adaptation generalizes preferentially between types of conflict with overlapping characteristics. However, although these between-dimension Graton effects occurred in the absence of confounds with the sequences of discriminative stimulus and response features, they were confined to abstract match trials. Thus, the results of Experiment 1 conform to the predictions of the semantic generalization hypothesis. By contrast, the conflict adaptation hypothesis does not seem to offer an explanation for this pattern of findings.

Although we intermixed blocks of trials in which participants responded to the direction of the stimulus arrow, direction-based interference (in the critical blocks) was overall small–considerably smaller than location-based interference and smaller than in the study of Kunde and Wühr (2006). Given the possibility that attentional adaptation correlates with conflict strength (e.g., Wendt et al., 2014), it may not have occurred in a detectable way under these conditions. To investigate the modulation of the congruency effects under conditions in which incongruent arrow distractors were likely to yield substantial conflict, we conducted a second experiment in which we used a task that involved the identification of the pointing direction of a target stimulus as left or right.

## Experiment 2

In Experiment 2 participants responded to a triangle that pointed either to the left or to the right by pressing the spatially corresponding key. Directional distractor information was presented in the form of a pair of different triangles, in which the target stimulus was embedded, forming a vertical target-flanker configuration. Like in Experiment 1, the stimuli could be presented in a left-sided or in a right-sided screen location. Experiment 2 thus combined location-based interference with interference evoked by spatially adjacent flanker stimuli. Noteworthily, previous studies that combined location-based interference and flanker interference, however in the context of non-spatial tasks, failed to obtain between-dimension Gratton effects (Stürmer et al., 2005; Wendt et al., 2006).

Although using left- and right-pointing triangles as target stimuli introduced additional overlap of left/right features, this did not change any contingencies of relevance for our analyses. This can be illustrated by replacing, in Table 2, white and black arrow color with left-and right-pointing target triangles, and left- and right-pointing arrows with left-and right-pointing flanker triangles, respectively. This would evidently not affect the contingencies of the sequences of congruency levels and the sequences of concrete as well as abstract distractor features and responses. Therefore, Experiment 2 could be analyzed along the same lines as Experiment 1.

### Method

#### Participants

Five female and 10 male students of the Helmut-Schmidt-University/University of the Federal Armed Forces Hamburg participated in exchange for partial course requirements. They ranged in age from 21 to 24 years.

#### Apparatus and stimuli

Apparatus and stimuli were the same as in the preceding experiments with the following exceptions. Arrow stimuli were replaced by a row of three, vertically aligned equilateral triangles, presented either 2.8 cm (medial edge) to the left or to the right of the screen center, and extending 5.5 cm vertically and 1.7 cm horizontally. The central (target) triangle was presented on the horizontal midline. Triangles had a side length of 1.8 cm and could point to the left or to the right. The same response keys were used as in Experiment 1.

#### Procedure

The procedure of Experiment 2 was the same as the procedure of Experiment 1 with the following exceptions. First, participants responded to the pointing direction of the central triangle by pressing the spatially corresponding response key. Second, on each trial, the pointing direction (left vs. right) of the central triangle as well as the pointing direction of the flanker triangles (left vs. right) were chosen randomly with the only constraint that the two distractor triangles pointed in the same direction. Figure 1 depicts schematic examples of stimulus displays used in different conditions. Third, after working through a practice block of 30 trials participants were administered 15 experimental (i.e., critical) blocks of 67 trials each. A complete session took between 45 and 60 min.

### Results

The same analyses and exclusion criteria were applied as in Experiment 1. RT outlier exclusion eliminated 0.04% of the data.

#### Overall analysis

Responses were slower when the current trial was associated with incongruent as compared to congruent flankers (486 vs. 415 ms), *F*_(1, 14)_ = 296.7; *p* < 0.01; *MSE* = 996.4. In contrast, there was no overall location-based congruency effect, *F*_(1, 14)_ < 1. Direction- and location-based interference interacted however, yielding a location-based congruency effect of 11 ms on direction-congruent trials and a slightly reversed location-based congruency effect of −5 ms on direction-incongruent trials, *F*_(1, 14)_ = 19.4; *p* < 0.01; *MSE* = 199.5. Within-dimension Gratton effects occurred for both dimensions. The direction-based congruency effect was reduced from 82 to 59 ms after a direction-incongruent compared to after a direction-congruent trial, *F*_(1, 14)_ = 32.9; *p* < 0.01; *MSE* = 252.6, and the location-based congruency effect was reversed from 37 ms after location-congruent to −31 ms after location-incongruent trials, *F*_(1, 14)_ = 98.0; *p* < 0.01; *MSE* = 715. The latter effect was further modulated by an interaction with direction-congruency of the preceding trial, *F*_(1, 14)_ = 6.6; *p* < 0.03; *MSE* = 212.4, indicating that the sequential modulation was somewhat larger when the preceding trial involved incongruent flankers. The direction-based congruency effect was also reduced after location-incongruent trials compared to after location-congruent trials (65 vs. 76 ms), *F*_(1, 14)_ = 16.0; *p* < 0.01; *MSE* = 113.4. This between-dimension Gratton effect was further modulated by an interaction with location-congruency of the current trial, *F*_(1, 14)_ = 39.2; *p* < 0.01; *MSE* = 148.1, indicating that the effect was confined to location-congruent trials. The location-based congruency effect was not affected by the direction-congruency level of the preceding trial (1 vs. 4 ms after direction-congruent and direction-incongruent trials, respectively), *F*_(1, 14)_ = 1.4; *p* = 0.25; *MSE* = 65.6.

In the error analysis, all main effects were significant, indicating that errors were more frequent with incongruent than with congruent flankers (4.1 vs. 1.3%), *F*_(1, 14)_ = 28.5; *p* < 0.01; *MSE* = 0.00163, and with incongruent than with congruent stimulus locations (3.5 vs. 1.9%), *F*_(1, 14)_ = 12.9; *p* < 0.01; *MSE* = 0.00116, and less frequent after a trial with incongruent than congruent flankers (2.4 vs. 3.0%), *F*_(1, 14)_ = 5.5; *p* < 0.04; *MSE* = 0.00042, as well as after a trial with an incongruent than with a congruent stimulus location (2.0 vs. 3.5%), *F*_(1, 14)_ = 14.9; *p* < 0.01; *MSE* = 0.00087. Direction- and location-based interference interacted overadditively, *F*_(1, 14)_ = 5.3; *p* < 0.04; *MSE* = 0.00068. Both the direction-based and the location-based congruency effect were reduced after a location-incongruent compared to a location-congruent predecessor trial, 1.9 vs. 3.6%, *F*_(1, 14)_ = 6.2; *p* < 0.03; *MSE* = 0.00075, and −1.4 vs. 4.5%, *F*_(1, 14)_ = 30.6; *p* < 0.01; *MSE* = 0.00172, respectively. A three-way interaction involving location-congruency of the current and of the preceding trial and direction-congruency of the current trial, *F*_(1, 14)_ = 13.3; *p* < 0.01; *MSE* = 0.00062, indicated that the location-based Gratton effect was more pronounced on trials with incongruent than with congruent flankers. Neither direction- nor location-based interference was affected by the direction-congruency level of the preceding trial, both *F*s < 1.

#### Semantic generalization effects

**Figure 4:**
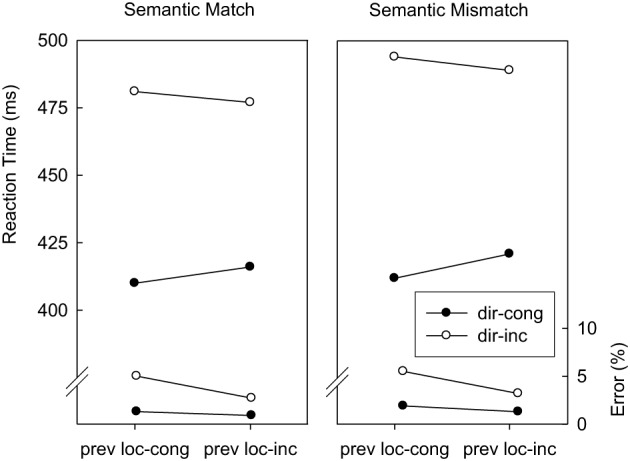
**Mean reaction times and error percentages in Experiment 2 as a function of direction-congruency on the current trial (dir-cong, dir-inc), location-congruency on the preceding trial (prev loc-cong, prev loc-inc), and correspondence of the abstract feature sequences (match, mismatch)**.

***Location-congruency (*N* − 1) → direction-congruency (N)***. Trials were classified as a function of direction-congruency in trial *N* (congruent, incongruent), location-congruency in trial *N* − 1 (congruent, incongruent), and abstract correspondence (match, mismatch). Figure 4 displays mean RTs and error percentages for these data. In RTs, the main effect of direction-congruency and the interaction with location-congruency on the preceding trial were replicated from the overall analysis, *F*_(1, 14)_ = 294.2; *p* < 0.01; *MSE* = 504.2, and *F*_(1, 14)_ = 14.7; *p* < 0.01; *MSE* = 85.6, respectively. Abstract mismatch trials were associated with an increase in overall RTs of 8 ms, *F*_(1, 14)_ = 22.1; *p* < 0.01; *MSE* = 95.5, and with an increase in direction-based interference of 8 ms, *F*_(1, 14)_ = 5.4; *p* < 0.04; *MSE* = 108.5. There was no sign of a three-way interaction involving direction-congruency of the current trial, location-congruency of the preceding trial, and abstract correspondence, *F*_(1, 14)_ = 1.2; *p* = 0.30; *MSE* = 32.1. The error analysis replicated the main effects of direction-congruency and preceding location-congruency as well as the interaction between these two factors from the overall analysis, *F*_(1, 14)_ = 30.0; *p* < 0.01; *MSE* = 0.00077, *F*_(1, 14)_ = 13.4; *p* < 0.01; *MSE* = 0.00045, and *F*_(1, 14)_ = 7.2; *p* < 0.02; *MSE* = 0.00033. Mirroring the RT results, the reduction of the direction-based congruency effect after location-incongruent trials was not modulated by abstract correspondence, *F*_(1, 14)_ < 1.

**Figure 5:**
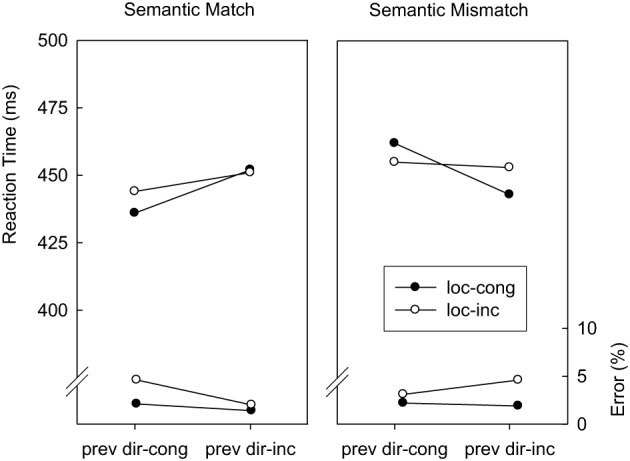
**Mean reaction times and error percentages in Experiment 2 as a function of location-congruency on the current trial (loc-cong, loc-inc), direction-congruency on the preceding trial (prev dir-cong, prev dir-inc), and correspondence of the abstract feature sequences (match, mismatch)**.

***Direction-congruency (*N* − 1) → Location-congruency (N)***. Trials were classified as a function of location-congruency in trial *N* (congruent, incongruent), direction-congruency in trial *N* − 1 (congruent, incongruent), and abstract correspondence (match, mismatch). Figure 5 displays mean RTs and error percentages for these data. In the RT analysis, the only significant main effect was abstract correspondence, *F*_(1, 14)_ = 11.9 *p* < 0.01; *MSE* = 141.2, indicating that abstract mismatch trials were associated with longer RTs than abstract match trials (453 vs. 446 ms). In addition, incongruent flankers in the preceding trial slowed responding on abstract match trials by 12 ms and speeded up responding on abstract mismatch trials by 10 ms, *F*_(1, 14)_ = 31.7; *p* < 0.01; *MSE* = 110.0. As in the overall analysis, the location-based congruency effect was not significantly affected by the direction-congruency level of the preceding trial, *F*_(1, 14)_ = 3.1; *p* = 0.10; *MSE* = 44.4. However, there was a significant three-way interaction, *F*_(1, 14)_ = 14.7; *p* < 0.01; *MSE* = 83.7. As can be seen in Figure 5, this was because on abstract match trials the location-based congruency effect was larger after direction-congruent than direction-incongruent trials, whereas on abstract mismatch trials the location-based congruency effect was larger after direction-incongruent than after direction-congruent trials. The error analysis replicated the main effect of location-based congruency from the overall analysis, *F*_(1, 14)_ = 13.6; *p* < 0.02; *MSE* = 0.00060. Also, the main effect of direction-congruency on the preceding trial approached significance, *F*_(1, 14)_ = 3.8; *p* = 0.07; *MSE* = 0.00024. Contrary to the RT analysis, incongruent flankers in the preceding trial decreased the error rate on abstract match trials by 1.7% and increased the error rate on abstract mismatch trials by 0.6%, *F*_(1, 14)_ = 15.8; *p* < 0.01; *MSE* = 0.00025. Although there was no overall reduction of location-based interference after direction-incongruent trials, *F*_(1, 14)_ < 1, a significant three-way interaction indicated that the location-based congruency effect was larger after direction-congruent than direction-incongruent trials on abstract match trials, whereas it was larger after direction-incongruent than after direction-congruent trials on abstract mismatch trials, *F*_(1, 14)_ = 8.2; *p* < 0.02; *MSE* = 0.00033.

### Discussion

Barring minor procedural differences Experiment 2 differed from Experiment 1 in that the target stimulus features were perceptually similar to the direction distractor features. As expected, the direction-based congruency effect was considerably larger under these conditions than in Experiment 1. In contrast to this enhancement of distractor interference, the main effect of location-based interference failed to reach significance in RTs.

Despite the absence of a significant overall effect of location-based interference, however, a clear-cut pattern of sequential modulations of congruency effects occurred. Again, we found within-dimension Gratton effects for both distractor dimensions, which cannot unequivocally be attributed to conflict adaptation because of the confound with distractor-target or distractor-response sequences. Replicating the findings of Experiment 1, a between-dimension Gratton effect was found for direction-based interference, and—deviating from the results of Experiment 1—displayed comparable strength for abstract match and mismatch trials. As such, these findings seem to provide novel evidence for the conflict adaptation account. However, the modulations of location-based interference pose a problem for this interpretation. Specifically, although there was no overall modulation of the location-based congruency effect by the direction-based congruency level of the preceding trial, this absence resulted from a between-dimension Gratton effect on abstract match trials and a reversed between-dimension Gratton effect (i.e., a larger congruency effect after an incongruent than after a congruent predecessor trial) on abstract mismatch trials. Viewed from the conflict adaptation perspective, the latter effect would suggest that location-congruent rather than location-incongruent trials increased attentional focusing. There seems to be no straightforward reason for such an assumption.

By contrast, the semantic generalization hypothesis offers a plausible explanation for the co-occurrence of a between-dimension Gratton effect for one of the distractor dimensions and a reversed between-dimension Gratton effect for the other distractor dimension, on abstract mismatch trials. As noted in the Introduction, on abstract mismatch trials conjoined repetitions/alternations regarding the response and the location-to-direction sequence are associated with partial repetitions regarding the response and the direction-to-location sequence and vice versa. We argued that, in the case that both abstract feature sequences yield equally strong effects, this arrangement should result in a null effect on abstract mismatch trials, as found in Experiment 1. On the other hand, there is no a priori reason to assume that the activation of a stored location code by perceiving a corresponding arrow direction is about as strong as the activation of a stored direction code by perceiving a stimulus in a corresponding location. Therefore, it is also conceivable that one of the assumed mechanisms, activation of a location code by a perceived direction or activation of a direction code by a perceived location, is substantially stronger than the other one, depending on yet unidentified stimulus and task characteristics.

Consider the case that the activation of a location code elicited by perceiving a corresponding arrow direction is stronger than the activation of a direction code elicited by perceiving a corresponding stimulus location. For abstract match trials, in which effects of the location-direction sequence and the direction-location sequence work in the same direction, this would lead to qualitatively the same expectation as an equal strength assumption. The prediction would be different, however, for abstract mismatch trials. Performance on these trials should be more affected by the location-direction sequence than by the direction-location-sequence. Looking at the abstract mismatch trials in Table 2 shows that between-dimension congruency level repetition (i.e., location-congruent → direction-congruent and location-incongruent → direction-incongruent) are associated with advantageous conjunctions (i.e., conjoined repetitions or alternations) of the abstract location-to-direction feature and the response. By contrast, between-dimension congruency level alternations (i.e., location-incongruent → direction-congruent and location-congruent → direction-incongruent) are associated with disadvantageous conjunctions (i.e., partial repetitions) of the abstract location-to-direction feature and the response. This pattern is perfectly reversed for sequences regarding the abstract direction-to-location feature and the response. Assuming that the direction-location sequences are less influential than the location-direction sequences, we would expect facilitation of direction-congruent trials after location-congruent trials and facilitation of direction-incongruent trials after location-incongruent trials. Hence we would expect direction-based interference to be reduced after location-incongruent trials also on abstract mismatch trials.

Regarding location-based interference after direction-congruent and direction-incongruent trials, however, the assumption that direction-location sequences are less influential than location-direction sequences would lead to the opposite prediction for abstract mismatch trials, that is increased location-based interference after direction-incongruency, as compared to after direction-congruency. This is because on such trials between-dimension congruency level repetitions are associated with partial repetitions regarding the abstract location-direction feature and the response, whereas between-dimension congruency level alternations are associated with partial repetitions regarding the abstract location-direction feature and the response. (This assertion can be verified by identifying the respective trial transitions in the shaded area of Table 2).

An analogous reasoning shows that if direction-to-location sequences had a larger impact than location-to-direction sequences, the semantic generalization hypothesis would predict location-based interference to be reduced after direction-incongruent trials on both abstract match and mismatch trials. Direction-based interference, on the other hand, should be reduced after location-incongruent trials on abstract match trials but increased on abstract mismatch trials.

In more general terms, the semantic generalization hypothesis predicts one of three different patterns regarding between-dimension sequential congruency modulations on abstract mismatch trials: no between-dimension Gratton effects at all (as found in Experiment 1), reduced direction-based interference after location-incongruent trials and increased location-based interference after direction-incongruent trials (as found in Experiment 2), or reduced location-based interference after direction-incongruent trials and increased direction-based interference after location-incongruent trials, depending on the relative strengths of “location by direction” and “direction by location” activation. The results of Experiment 2 are consistent with the semantic generalization hypothesis if it is assumed that activation of a location code by perceiving a corresponding arrow outweighs activation of a direction code by perceiving a corresponding stimulus location under the conditions set up in the experiment.

## General discussion

The present article deals with trial-to-trial modulations of interference evoked by processing a distractor stimulus feature, focusing on reductions of interference evoked by one distractor dimension after conflict evoked by a perceptually different distractor dimension. The presence or absence of such between-dimension Gratton effects bears theoretical importance for two reasons. First, the specific pattern of distractor dimensions for which between-dimension Gratton effects are found may provide insights regarding the specific processes of conflict detection and adaptation. Second, findings of between-dimension Gratton effects with an experimental set-up like the one realized in the current study might provide valuable evidence for the notion of attentional conflict adaptation as such. This is because so far within-dimension Gratton effects could not be replicated in conditions of rigorous control of feature sequence and distractor-target/response contingencies unless the task used was divided into a pair of non-overlapping two-choice tasks (Freitas and Clark, 2014; Kim and Cho, 2014; Schmidt and Weissman, 2014; Weissman et al., 2014). Investigations of between-dimension Gratton effects offer a different method of feature sequence control by including, with equal probability, data from trials with all kinds of feature repetitions and alternations in all congruency level sequences. Although the use of a single two-choice task precludes considering only complete feature change trials, observing Gratton effects under these conditions would broaden the empirical basis for the idea of attentional conflict adaptation.

In the current study, we used lateralized responses and combined interference evoked by left- vs. right-sided presentation of the stimulus and by stimulus shapes pointing to the left or right. Distractor dimensions involving left/right variations have been combined in at least three previous studies in which between-dimension Gratton effects were observed. First, as described in detail in the Introduction, Kunde and Wühr (2006, Experiment 2) used arrow stimuli, pointing to the left or right, presented at a left- or right-sided location. Similarly, Freitas and Clark (2014) combined different versions of a left-right spatial Stroop task with a flanker task comprising left-pointing vs. right-pointing arrows or pictures of left-pointing vs. right-pointing hands (Experiment 2), as well as a “Trajectory Stroop” task with a flanker task which again both comprised distractor stimuli pointing in one of two possible directions (Experiments 3A and B). Finally, Notebaert and Verguts (2008) used left- and right-sided stimulus presentation and SNARC (Spatial Numerical Association of Response Codes) correspondence (Dehaene et al., 1993). In that study, participants made lateralized key presses to lateralized Xs as well as to centrally presented digits. The SNARC effect is characterized by facilitation of left-sided responses when the value of a to-be-classified digit is small and of right-sided responses when the value of a to-be-classified digit is large. Thus, the two distractor dimensions overlapped semantically on the left/right dimension. Between-dimension Gratton effects were found, albeit this was confined to a situation in which both digits and Xs required the same type of judgment (i.e., presentation format normal vs. italics, condition 1) and did not occur when the Xs required a different judgment (i.e., color, condition 2).

In light of the fact that a considerable number of previous studies have failed to obtain between-dimension Gratton effects, the results of the experiments of the current study (i.e., between-dimension Gratton effects for three of four comparisons) accords with the assumption that semantic overlap between distractor dimensions plays a facilitative role in generating such effects. It is conceivable that semantic overlap of distractor dimensions promotes representing both dimensions in a linked structure, thereby possibly enhancing the likelihood of generalized conflict adaptation. Although we cannot dismiss this possibility, an alternative explanation seems better suited to account for the overall pattern of results we obtained. Specifically, we hypothesized that overlap between abstract stimulus features may result in activation of a stimulus feature code in episodic memory by perception of a semantically related feature of the other dimension. With this assumption it is possible, in principle, to account for between-dimension Gratton effects in terms of processing disadvantages on between-dimension congruency level alternations due to partial repetitions regarding the responses and abstract stimulus features.

Consistent with this hypothesis, the between-dimension Gratton effects in Experiment 1 were confined to abstract match trials, whereas there was no trial-to-trial modulation of the congruency effect on abstract mismatch trials, a result difficult to explain by the assumption of (dimension-unspecific) conflict adaptation. In Experiment 2, the task required responding to the direction of a target stimulus. Although this manipulation did not alter the sequences of concrete and abstract distractor and response features, it added perceptual overlap of the target stimulus dimension with the direction distractor dimension (and also semantic overlap of the target stimulus dimension with both distractor dimensions). This arrangement resulted not only in an overall larger congruency effect evoked by the direction distractor but also yielded a more complicated pattern of between-dimension Gratton effects. Precisely, whereas a between-dimension Gratton effect, unaffected by the abstract feature sequence, occurred for the direction dimension, the location dimension was associated with a between-dimension Gratton effect for abstract match trials but a reversed Gratton effect for abstract mismatch trials. Viewed from a conflict adaptation perspective, this pattern of results would suggest that attentional focusing was stronger after (direction-) congruent than after (direction-) incongruent trials on abstract mismatch trials. There seems to be no straightforward reason for this assumption. By contrast, the semantic generalization hypothesis offers a plausible explanation for this pattern of findings.

Specifically, considering the possibility of different strengths of abstract feature sequence effects on location-direction and direction-location transitions, three different patterns of a three-way interaction of congruency of the current trial, congruency regarding the other distractor dimension on the preceding trial, and the abstract feature sequence (match vs. mismatch) are conceivable. The results obtained in Experiments 1 and 2 constitute one of them each. Given the absence of an independent measure of relative strength of abstract feature sequence effects, this interpretation can only be applied with caution. On the other hand, because all of the three possible result patterns involve a modulation of the sequential congruency effect by the sequence of abstract stimulus and response features, they seem difficult to account for in terms of conflict adaptation. Further research regarding semantic generalization of feature integration effects could be undertaken by means of manipulating semantic overlap between prime and probe stimuli in Hommel's feature integration paradigm (e.g., Hommel, 1998; Hommel and Colzato, 2004). Such manipulations may be useful, in particular, to explore possible strength asymmetries.

As noted in the Introduction, recent studies have provided strong evidence for conflict adaptation, uncontaminated by feature repetitions and biased distractor-target/response contingencies. Investigating sequential congruency effects evoked by different perceptual dimensions complement this approach and seem particularly useful to examine the question of specificity vs. generality of conflict adaptation. Providing initial evidence for semantic generalization effects of stimulus and response feature integration, however, our current study demonstrates a limitation in attributing between-dimension Gratton effects to generalized conflict adaptation if the two distractor dimensions overlap semantically. Such inference seems justified only if between-dimension Gratton effects are consistently found not only for abstract match trials but also for abstract mismatch trials, for both distractor dimensions that are combined in the experiment.

### Conflict of interest statement

The authors declare that the research was conducted in the absence of any commercial or financial relationships that could be construed as a potential conflict of interest.

## Footnotes

^1^Contrasting with the between-dimension sequential congruency manipulation, none of the above studies controlled for feature sequence and contingencies regarding within-dimension sequential congruency effects. Inferring dimension-specificity of conflict adaptation from these studies (e.g., Egner et al., 2007; Schlaghecken et al., 2011; Lee and Cho, 2013) may thus be premature because the within-dimension Gratton effects found might reflect feature integration or contingency level switching.

^2^Whereas in the experiment of Kunde and Wühr (2006) participants were instructed to identify the direction of a target arrow stimulus, Table 2 two depicts a situation in which stimulus color acts as the target feature, thereby avoiding overlap with the distractor stimulus dimensions. This difference is not relevant regarding the relationship of consecutive congruency levels and distractor/response feature sequences displayed in the table.
